# Unraveling the spatial–temporal distribution patterns of soil abundant and rare bacterial communities in China’s subtropical mountain forest

**DOI:** 10.3389/fmicb.2024.1323887

**Published:** 2024-02-12

**Authors:** Panpan Wu, Dandan Hu, Jiaheng Guo, Jinlong Li, Quanlin Zhong, Dongliang Cheng

**Affiliations:** ^1^Institute of Geography, Fujian Normal University, Fuzhou, Fujian, China; ^2^Fujian Provincial Key Laboratory of Plant Ecophysiology, Fujian Normal University, Fuzhou, China; ^3^Key Laboratory of Humid Subtropical Eco-geographical Process, Ministry of Education, Fuzhou, China

**Keywords:** abundant bacteria, elevation, rare bacteria, season, soil physico-chemical properties, subtropical mountain ecosystems

## Abstract

**Introduction:**

The pivotal roles of both abundant and rare bacteria in ecosystem function are widely acknowledged. Despite this, the diversity elevational patterns of these two bacterial taxa in different seasons and influencing factors remains underexplored, especially in the case of rare bacteria.

**Methods:**

Here, a metabarcoding approach was employed to investigate elevational patterns of these two bacterial communities in different seasons and tested the roles of soil physico-chemical properties in structuring these abundant and rare bacterial community.

**Results and discussion:**

Our findings revealed that variation in elevation and season exerted notably effects on the rare bacterial diversity. Despite the reactions of abundant and rare communities to the elevational gradient exhibited similarities during both summer and winter, distinct elevational patterns were observed in their respective diversity. Specifically, abundant bacterial diversity exhibited a roughly U-shaped pattern along the elevation gradient, while rare bacterial diversity increased with the elevational gradient. Soil moisture and N:P were the dominant factor leading to the pronounced divergence in elevational distributions in summer. Soil temperature and pH were the key factors in winter. The network analysis revealed the bacteria are better able to adapt to environmental fluctuations during the summer season. Additionally, compared to abundant bacteria, the taxonomy of rare bacteria displayed a higher degree of complexity. Our discovery contributes to advancing our comprehension of intricate dynamic diversity patterns in abundant and rare bacteria in the context of environmental gradients and seasonal fluctuations.

## 1 Introduction

Soil bacteria are essential drivers of biogeochemical processes and productivity of terrestrial ecosystem (Saleem et al., 2019; Hou et al., 2020). Moreover, the soil bacterial communities exhibited substantial rare species and few abundant species (Dong et al., 2021; Wang et al., 2021a). In terrestrial ecosystems, abundant species are prominent members involved in ecosystem nutrient cycling (Kurm et al., 2019). Additionally, rare species, despite their low abundance, may exhibit heightened metabolic activity relative to abundant taxa under similar environmental conditions (Jousset et al., 2017; Xue et al., 2018). Moreover, they can function as keystone species, performing a crucial function in regulating ecosystem functions (Singh B. K. et al., 2014; Zhang et al., 2020). Hence, it is apparent that both abundant and rare bacteria assume the primary role in regulating ecosystem functions. To date, numerous studies have been conducted on abundant and rare bacterial communities in divergent ecosystems (Du et al., 2020; Wang et al., 2021b; Zhao et al., 2022; Zhang et al., 2023). However, inconsistent results have been reported regarding the community diversities and driving factors of abundant and rare bacteria (Hou et al., 2020; Su et al., 2022). Therefore, comparing the distribution patterns and drivers of soil abundant and rare bacterial community diversities in mountain ecosystems may provide more insightful knowledge about soil bacterial communities.

Abundant and rare bacteria exhibit different responses to soil factors, leading to distinct distribution patterns (Zeng and An, 2021; Wang et al., 2021a). Soil pH is widely recognized as a crucial variable influencing the diversity of soil bacterial communities (Ni et al., 2021; Zou et al., 2023). The diversity of abundant bacteria peaks at a moderate pH, while the diversity of rare bacterial communities increases in more acidic and alkaline soils (Hou et al., 2020). Additionally, changes in temperature are likely to be a better predictor of bacterial community diversity than soil pH (Zhou et al., 2016). Temperature can affect soil water and nutrient availability (Wang J. et al., 2017), with the abundance of abundant bacteria increasing significantly with rising temperature, while rare bacteria show the opposite trend in temperate desert regions (Wang et al., 2021a). Soil properties exert a substantial influence on abundant and rare bacterial distribution (Ji et al., 2020), but its relative impacts vary depending on the geographic scale and ecosystem type. For example, within agricultural ecosystems, soil pH predominantly drives the composition of the abundant bacterial community, while temperature plays a essential role in formation of these rare bacteria (Jiao and Lu, 2020). Additionally, the abundant bacterial community is primarily influenced by soil organic carbon (C), pH and plant richness, while the factors affecting rare bacterial communities are soil nitrogen (N) concentration and pH in the Tibetan Plateau (Jiang et al., 2019). In summary, the diversity generation and maintenance mechanisms exhibit notable differences between abundant and rare bacterial communities, which indicates that they may have different responses to potential future climate fluctuations (Jiang et al., 2019).

Understanding how biodiversity responds to elevational gradients provides valuable insights into comprehending how climate change affects ecosystems (Li et al., 2017). The contrasting ecological functions of abundant and rare bacterial groups contribute to their varying sensitivities and adaptive capacities in the face of changing environmental conditions. Abundant species demonstrate a broader capacity for adaptation across a wide range of environmental gradients. In contrast, rare bacteria exhibit a closer relationship to specific environmental changes, such as variations in soil temperature and pH (Wan et al., 2021). More importantly, the association between elevational gradients and variation in specific environmental parameters, such as temperature and nutrient content, has been well-documented (Li et al., 2020). These environmental variables play a pivotal function in forming the both abundant and rare bacterial communities and their respective diversity (Jiao and Lu, 2020). Consequently, distribution trends of abundant and rare bacterial diversity may vary prominently along elevations. However, it is significant to note that most studies on elevational gradients have primarily focused on total soil bacterial communities (Ren et al., 2021; Tian et al., 2023), and the patterns of soil bacterial diversity have varied, including instances of increase (Duan et al., 2021), decrease (Zhang et al., 2022), and hump-shaped patterns (Tian et al., 2021). Only limited research has explored the trends of abundant and rare bacteria along elevational gradients, and these studies have been primarily conducted in aquatic ecosystems (Li et al., 2017; Liu et al., 2019). Thus, additional studies addressing the changing patterns and drivers along elevational gradients of these two bacterial diversities in montane ecosystems are essential for a comprehensive understanding of the elevational patterns of microbial diversity.

Apart from the elevational gradients, season can influence the bacterial community through various mechanisms, including the effects of plant photosynthesis. Seasonal changes in plant activity, such as litter fall and nutrient absorption, can impact bacterial communities by providing organic matter and altering nutrient availability in the soil. Moreover, seasonal variations of soil properties (e.g., temperature and moisture) could directly influence bacterial community composition and activity (Bardgett et al., 2005). Soil moisture is a decisive factor that profoundly influences microbial physiological activity by influencing soil physical structure and nutrient effectiveness (Li et al., 2021). The seasonal fluctuation in soil moisture can exert a significant impact on the total soil microbial community (Barnard et al., 2013). However, the microcosm experiment with three wetting-drying cycles demonstrated that temporal factors, specifically the number of cycles, exerted a more prominent influence on the abundance and diversity of both abundant and rare bacteria compared to soil moisture (Li et al., 2021). Furthermore, rare bacteria in subtropical region were not significantly influenced by seasonal precipitation variation (Zhao et al., 2017). Temperature exerts a direct control on the acceleration of metabolic rates and biochemical processes, making it a primary driving factor for all biological processes (Gillooly et al., 2001). Therefore, considering the influence of seasonal temperature changes is vital for understanding the responses of abundant and rare bacterial communities in various ecosystems. Despite accumulation of some research in the field, there remains a substantial gap in knowledge of the dynamics of both these bacterial communities along elevational gradients and their responses to seasonal variations and soil physico-chemical properties. This knowledge gap is particularly evident in subtropical mountain ecosystems characterized by distinct elevational gradients and pronounced variations in temperature and precipitation between winter and summer. Therefore, further investigations are warranted to explore and unravel the intricate associations between bacterial communities, environmental parameters, and ecosystem dynamics in these specific subtropical mountain ecosystems.

In present study, we intend to survey the distribution trends of both abundant and rare bacterial communities across a wide range of elevations, ranging from 430 to 2,100 m, in a field experiment conducted at Wuyi Mountain, China. Additionally, we will explore the soil factors that drive the observed patterns of bacterial community distribution. This primary objective of this study is to survey the impact of soil factors at different elevations and seasons on the composition and dynamic of these two communities. By examining the microbial diversity, community structure, and co-occurrence patterns, we aim to build a deeper knowledge of how these aspects of microbial ecology responds to global climate change. Specifically, this study is designed to address two fundamental objectives that are crucial for understanding bacterial community dynamic in subtropical mountain ecosystem: (1) Uncovering the change patterns of diversity in abundant and rare bacterial communities along elevational gradients in different seasons is the focus of this study. Given that abundant and rare taxa possess completely divergent ecological functional traits (Liang et al., 2020; Wan et al., 2021), we hypothesized that abundant and rare bacterial diversity would exhibit differences, or even contradictions, along the elevational gradient. (2) Identifying the primary factors influencing the distribution of these two communities in subtropical mountain ecosystems. In general, rare taxa are more sensitive to environmental change than abundant taxa (He J. et al., 2023), we hypothesized that soil properties would exert a significant influence on the elevational patterns of these two bacterial communities; however, soil properties would have a more significant impact on the rare bacterial community compared to abundant bacteria. It will offer insightful information on the ecological mechanisms and processes governing the distribution of abundant and rare bacteria in a context of changing environmental conditions by addressing these fundamental questions.

## 2 Materials and methods

### 2.1 Study location

The study area was located in Wuyi Mountain National Park, China (27°48′11″-28°00′35” N, 117°39′30″–117°55′47″ E). This region is characterized by a subtropical central monsoon climate zone, with elevations ranging from 350 to 2160.8 m. The average annual temperature in the area ranges from 13.2 to 14.8°C. As for precipitation, the average annual rainfall in the zone is reported to be within the range of 1813–3,544 mm. In August and December 2020, four specific elevational gradients (430, 1,200, 1700, and 2,100 m) were selected. At each of these elevational gradients, three 20 × 20 m plots were established with a separation distance of more than 20 m between plots. Soil temperature (ST) was monitored using a TMS temperature and humidity recording instrument (TMS-5) in each plot at the four elevational gradients.

### 2.2 Soil sampling and analysis

After removing surface litter layer, 0–15 cm soil was collected using the five-point method (Dear et al., 1992) in each sample plot. In total, 80 soil samples (4 elevational gradients × 2 seasons × 10 replicates) were collected in this experiment. Soil physico-chemical properties were determined following standard protocols. The gap in weight between dry and fresh soil after being dried to a weight that remains at 105°C is referred to as soil moisture (SM). The pH was detected by testing a 1:2.5 of air-dried soil to water mixture applying a pH meter (STARTER 300, Ohaus, USA). Soil total C (TC), N (TN) and P content (TP) were determined using air-dried soil crushed with a grinder after passing through a 100-mesh sieve. TP was measured through digesting samples with H_2_SO_4_-HClO_4_ (4,1). TC and TN were measured by a CHNOS elemental analyzer (Elementar Vario EL III, Elementar, Germany). Soil NH_4_^+^-N and NO_3_^−^-N content were extracted using a 2 mol·L^−1^ KCl solution at a ratio of 1:4. Dissolved organic C (DOC) and N (DON) were extracted using deionized water (1,4 fresh soil, water). The soil-water mixture was shaken for 30 min to facilitate the extraction of organic carbon and nitrogen compounds. The extracted solutions were then measured using a TOC analyzer (TOC-VCPH/CPN, Shimadzu, Japan). The continuous flow analyzer (San + +, Skalar, Netherlands) were used to detected NH_4_^+^-N, NO_3_^−^-N, TP and DON.

### 2.3 Soil bacterial community analysis

We extracted soil genomic DNA using the DNeasy® PowerSoil® Pro Kit (QIAGEN, USA), and determined of the DNA extracts using a NanoDrop 2000 UV–Vis spectrophotometer (Thermo Scientific, Wilmington, USA). 338F and 806R primers were utilized for amplicon sequencing of the V3-V4 region of the 16S rRNA gene. Paired sequencing was performed on amplicons utilizing the MiSeq PE300 platform (San Diego, CA, USA). Raw data were upload to the NCBI database (Accession Number: SRP437273). The raw sequencing data were processed using fastp (Chen et al., 2018) and FLASH (Magoč and Salzberg, 2011). The Uparse method was employed to cluster sequences into operational taxonomic units (OTUs) based on the threshold of 97% similarity (Stackebrandt and Goebel, 1994; Edgar, 2013), and chimeric sequences were identified and removed. The 16S rRNA database (Silva v138) was analyzed for taxonomy with RDP classifier (Wang et al., 2007). Detailed methods of sequencing are provided in Supplementary Information. OTUs with a relative abundance >0.1% were considered “abundant” in one sample, while those with a relative abundance <0.01% were classified as “rare” in one sample (Zheng et al., 2021; Zhao et al., 2022). Among these OTUs, 4,322 OTU were classified as rare taxa, and 1,103 OTU were assigned as abundant taxa, respectively.

### 2.4 Statistical analysis

The calculation of abundant and rare bacterial α-diversities was performed by mothur software (v.1.30.2). We assessed data for compliance with homogeneity of variance and normality before conducting one-way ANOVA and t-tests. If the assumptions were not met, the Kruskal–Wallis test and the Wilcoxon rank-sum test were used to evaluate elevational and seasonal differences in α-diversities. The results were visualized based on the “ggplot2” R package in R 4.2.2. We constructed linear mixed-effects models (LMM), where elevation and season are fixed factors and block is a random factor, to evaluate whether elevation, season and their interactions have an effect on abundant and rare bacterial diversity.

We employed a Veen plot analysis to assess the shared and unique OTUs among total, abundant, and rare bacterial taxa across different elevations and seasons. To examine the community structure of the total, abundant and rare bacterial communities, principal coordinate analysis (PCoA) were employed based on Bray–Curtis dissimilarity matrices. Analysis of similarities (ANOSIM) were utilized to assess the significance of elevation and seasonal differences in community. Soil physico-chemical properties was selected by the variance inflation factor (VIF) analysis, and those exhibiting VIF >10 were excluded to mitigate the influence of multicollinearity (Hair, 2011). We first performed detrended correspondence analysis (DCA) to calculate the gradient length using species-sample data (OTU table with 97% similarity). If the first axis of gradient lengths was greater than or equal to 3.5, canonical correspondence analysis (CCA) was used. If it was less than 3.5, redundancy analysis (RDA) was adopted to assess the effects of soil physico-chemical properties on microbial communities (Shankar et al., 2017; Liu et al., 2023). The significant influencing factors were determined based on permutest analysis. A heatmap was created to visualize and assess the correlation between the top 5 most abundant taxa in the total, abundant and rare bacterial communities and soil properties. The above analyses and Phyla composition plot were done on Majorbio Cloud Platform^1^.

Modified normalized stochasticity ratio (MST) analysis based on the Bray-Curtis distance and the Jaccard distance was performed by using the NST package of the R software to quantitatively evaluate the relative importance of deterministic and stochastic assembly processes for abundant and rare bacterial community (Guo et al., 2018; Ning et al., 2019; Guo et al., 2021). The Wilcox test was used to compare whether there were significant differences in MST values between the two groups.

The interaction patterns between abundant and rare bacterial communities at each elevation in summer and winter were investigated using network analysis. Only the sample discovery rate of OTUs >20% was adopted in the analyses. First, we utilized the “rcorr” function in the “Hmisc” R package to get the Spearman correlation coefficient (ρ) between the two OTUs. The link between two OTUs was deemed robust when the ρ > 0.8 and FDR-corrected *p* < 0.01. Then we use the “igraph” R package to generate a network graph file in gml format, and import Gephi v. 0.9.2 to visualize the correlation network. The number of nodes and edges, the average path length, the average degree, the average clustering coefficient, and the modularity were calculated to characterize the topological properties of the generated network levels. The fast greedy modularity optimization algorithm was employed to identify distinct modules or communities within the network. We identify the topological roles played by specific network nodes based on the thresholds of Zi and Pi (Ling et al., 2016). The topological roles of network nodes are clustered into four categories:modular hubs (Zi > 2.5, Pi <0.62), network hubs (Zi > 2.5, Pi >0.62), connectors (Zi < 2.5 and Pi >0.62) and peripheral (Zi < 2.5, Pi <0.62). Based on the degree of connectivity between each network node, keystone species were found. The other three types except peripherals were usually keystone species (Deng et al., 2012; Banerjee et al., 2016).

## 3 Results

### 3.1 The variation of soil properties along the elevational gradients across different seasons

Elevation, season and their interaction have significant impact on soil physico-chemical properties (LMM, *p* < 0.05) (Supplementary Table S1). Most of soil physico-chemical properties differ remarkably along the elevational gradients across different seasons (*p* < 0.05) (Supplementary Table S2). For example, TC, TN, and TP showed a significant increase with increasing elevation at summer (One-way ANOVA, *p* < 0.05), TC at 1200 m was notably higher than that at 430, 1700 and 2,100 m in winter, TN at 1200 m was dramatically higher than that at 430 m in winter. TP increased notably with increasing elevation in winter (One-way ANOVA, *p* < 0.05). There were remarkable seasonal differences in the ST, NH_4_^+^-N, TC, TN, and TP at the same elevational gradient (*t*-test, *p* < 0.05).

### 3.2 Distributions patterns of soil abundant and rare bacterial diversity and community along an elevational gradient and across different seasons

**Figure 1:**
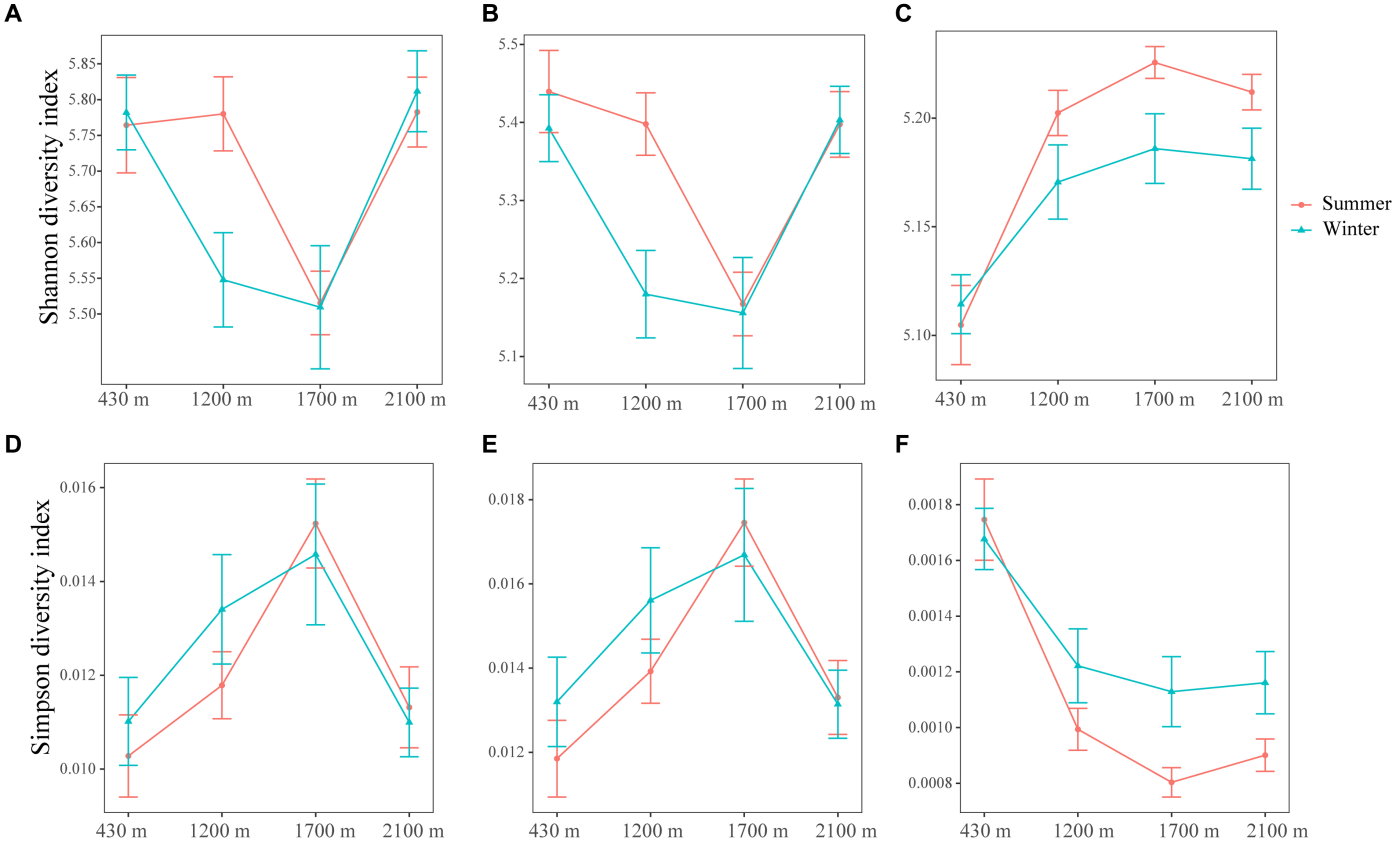
The line and symbol plot of soil bacterial α-diversities along elevational gradients. Shannon of total **(A)**, abundant **(B),** and rare **(C)** bacteria in soil along elevational gradients at different seasons, respectively. Simpson of total **(D)**, abundant **(E),** and rare **(F)** bacteria in soil along elevational gradients at different seasons. Values are means ± SE (*n* = 10). The red lines and error bars represent bacterial α-diversities in summer. The blue lines and error bars represent bacterial α-diversities in winter.

Elevation and season exert a profound influence on the rare bacterial diversity, with elevation exerting a stronger effect compared to seasonal variation (LMM, *p* < 0.001) (Supplementary Table S4). On the contrary, elevation, season and their interaction had no influence on abundant bacterial diversity (Supplementary Table S4). Here, the total and abundant bacterial diversity initially decreased and then increased with elevation, while rare bacterial diversity showed a consistent increase with increasing elevation (Figure 1). The Shannon and Simpson values of total, abundant and rare bacterial diversities exhibited similar change over with elevation in summer and winner (Figure 1). Specifically, At 1200 m, the Shannon index values of abundant bacteria in summer were markedly higher than in winter (t-test, *p* < 0.05) (Figure 1B and Supplementary Table S3). At 1700 m, the Simpson index values of rare bacteria in winter were dramatically more than in summer (Figure 1F and Supplementary Table S3), while Shannon index values was higher in summer (Figure 1C and Supplementary Table S3). Our findings illustrated that abundant and rare bacterial diversity were higher during the summer season compared to winter.

**Table 1:** Soil physico-chemical properties measured and their abbreviations.

Soil physico-chemical properties	Acronym	Unit
Soil moisture	SM	%
Soil temperature	ST	°C
Soil pH	pH	–
Soil ammonium nitrogen concentration	NH_4_^+^-N	mg·kg^−1^
Soil nitrate nitrogen concentration	NO_3_^−^-N	mg·kg^−1^
Soil total carbon concentration	TC	g·kg^−1^
Soil total nitrogen concentration	TN	g·kg^−1^
Soil total phosphorus concentration	TP	g·kg^−1^
The ratio of TC to TN	C:N	-
The ratio of TC to TP	C:P	-
The ratio of TN to TP	N:P	-
Soil dissolved organic-carbon concentration	DOC	mg·kg^−1^
Soil dissolved organic-nitrogen concentration	DON	mg·kg^−1^

**Figure 2:**
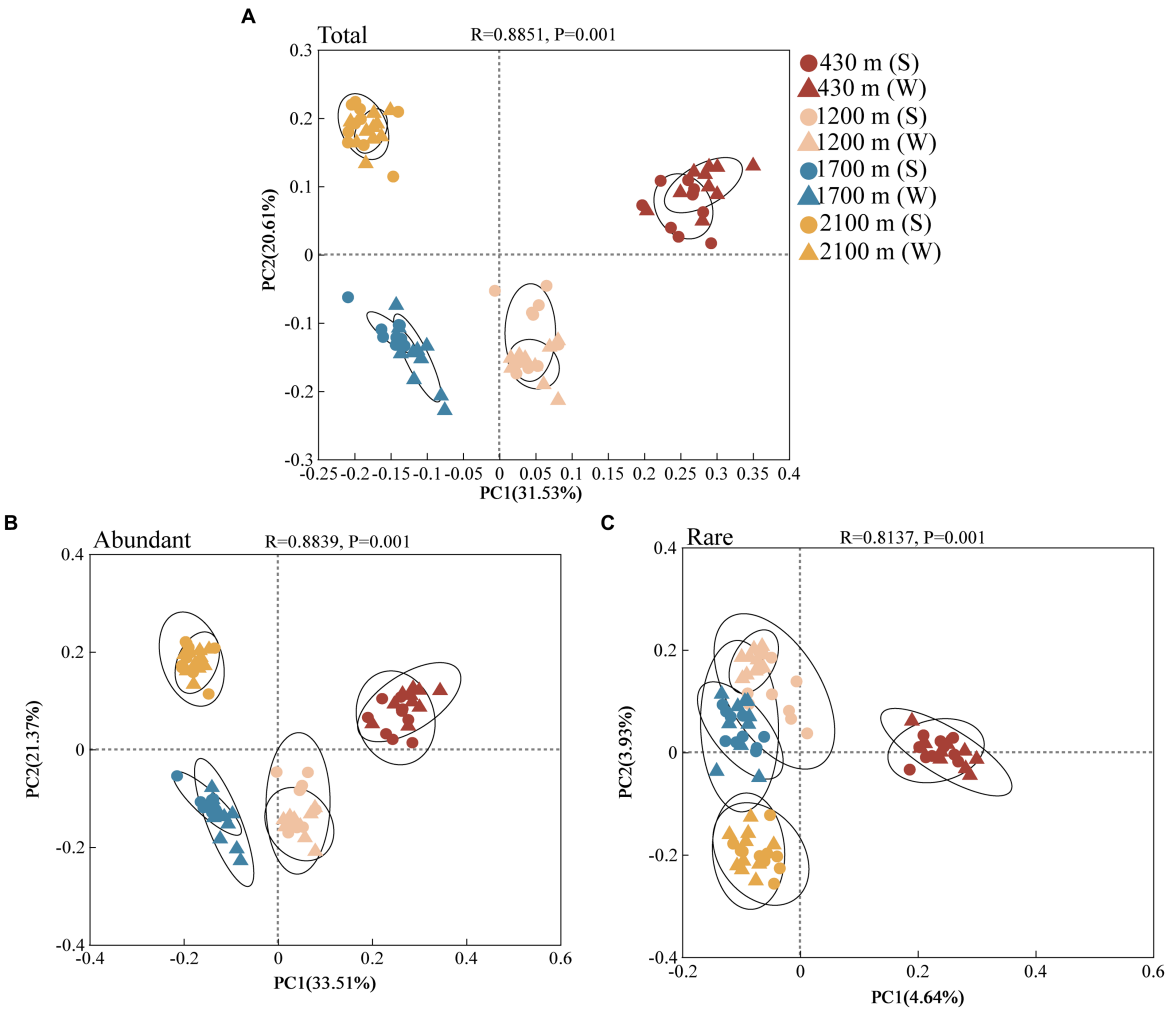
Total **(A)**, abundant **(B),** and rare **(C)** bacterial communities in soil at different elevational gradients and seasons identified by principal co-ordinates analysis (PCoA) at the OTU level (*n* = 80). The dots represent summer and triangles represent winter. Different colors represent 430, 1,200, 1700, and 2,100 m, respectively.

PCoA analysis indicated that components 1 and 2 explained 33.51 and 21.37% variation of abundant bacterial communities, respectively (Figure 2B). explained 4.64 and 3.93% variation of rare bacterial communities (Figure 2C). Analysis of similarities (ANOSIM) revealed significant elevational variations in the abundant and rare bacterial community during both the summer and winter seasons (Supplementary Table S6, *p* < 0.001). Under the same elevation gradient, these two communities also exhibited remarkable seasonal divergences, except for the rare bacterial communities at 2100 m, which did not show significant seasonal differences (Supplementary Table S7).

The rare bacteria display a larger number of taxonomic groups compared to the abundant bacteria (Supplementary Figure S1). The dominant phyla of total bacteria was Proteobacteria (Supplementary Figures S1A,B). The relative abundance of Planctomycetota exhibited a consistent variation with elevation within each season among the abundant bacterial taxa (Supplementary Figures S1C,D). Dependentiae, Bdellovibrionota, Patescibacteria, Bacteroidota, Cyanobacteria, Elusimicrobiota, Armatimonadota, and Gemmatimonadota were particular to rare bacterial taxa (Supplementary Figures S1E,F). The Veen plot analysis revealed that a substantial proportion (69.17%) of OTUs in abundant bacterial taxa were shared across all four elevations and both seasons. However, the percentage of shared OTUs for rare bacterial taxa (0.53%) was notably lower. Rare bacterial taxa showed a remarkable separation compared to abundant bacterial taxa (Supplementary Figure S2).

The MST ratio indicated that the abundant bacterial community in both summer and winter was predominantly driven by deterministic assembly processes (MST < 50%). Moreover, there was no significant difference in MST values between summer and winter (Supplementary Figure S3A), suggesting that deterministic processes exert nearly identical effects on abundant bacterial communities in both summer and winter. However, rare bacterial community were primarily influenced by stochastic processes (MST > 50%). Additionally, the MST values of rare bacterial communities in summer were significantly higher than those in winter (*p* < 0.05) (Supplementary Figure S3B), indicating that rare bacterial taxa in summer are more affected by dispersal limitations.

### 3.3 Driving factors influencing the distribution trends of both abundant and rare bacterial communities

The physico-chemical properties of the soil have a greater impact on rare bacterial diversity than on abundant bacteria. TN has larger effects on the rare bacterial diversity, while DOC and DON exhibit greater effects on abundant bacterial α-diversity. Bacterial diversity is found to be significantly influenced by SM and pH (Spearman rank correlation test, *p* < 0.05) (Supplementary Table S5).

**Figure 3:**
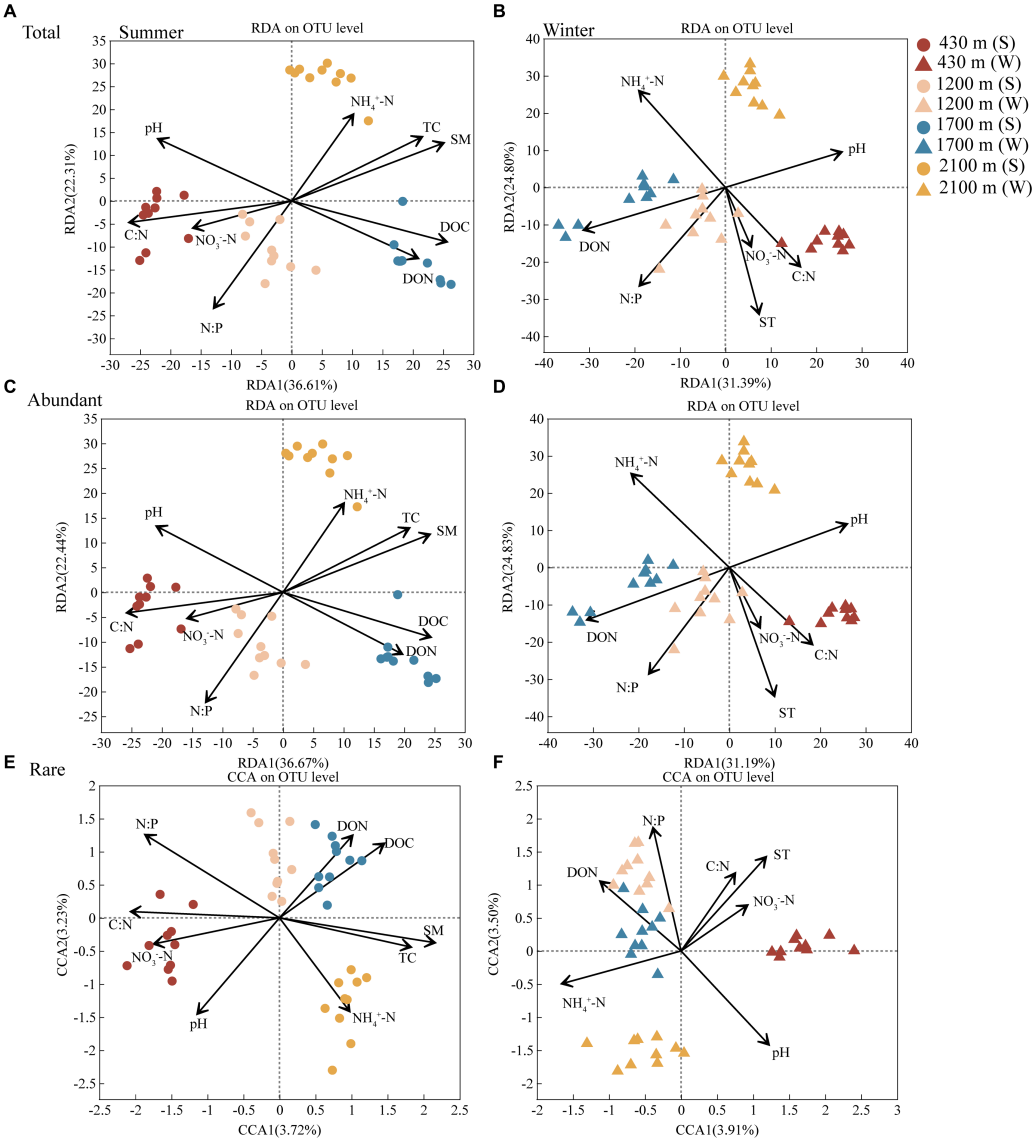
The redundancy analysis (RDA) or canonical correspondence analysis (CCA) plot showing the factors driving the total **(A)**, abundant **(C)**, and rare **(E)** bacterial communities in summer and the total **(B)**, abundant **(D)**, and rare **(F)** bacterial communities in winter, respectively (*n* = 80). Different colors represent 430, 1,200, 1,700, and 2,100 m, respectively. Dots represent summer and triangles represent winter. Abbreviations for soil physico-chemical properties are shown in Table 1.

Soil TC, N:P, C:N, pH, SM, NH_4_^+^-N, NO_3_^−^-N, DON, and DOC had remarkable effects on abundant and rare bacterial communities in summer. Meanwhile, SM (Permutest, *R*^2^ = 0.8368, *p* < 0.001) and N:P (*R*^2^ = 0.8094, *p* < 0.001) were the major factor contributing to the elevational difference in these two communities at summer, respectively (Figure 3 and Supplementary Table S8). ST, pH, NH_4_^+^-N, NO_3_^−^-N, C:N, N:P, DON were driving factors for abundant and rare bacterial communities in winter. Moreover, ST (*R*^2^ = 0.8034, *p* < 0.001) and pH (*R*^2^ = 0.8840, *p* < 0.001) were the main factor contributing to the difference in these two communities along elevational gradient in winter, respectively (Figure 3 and Supplementary Table S9).

**Figure 4:**
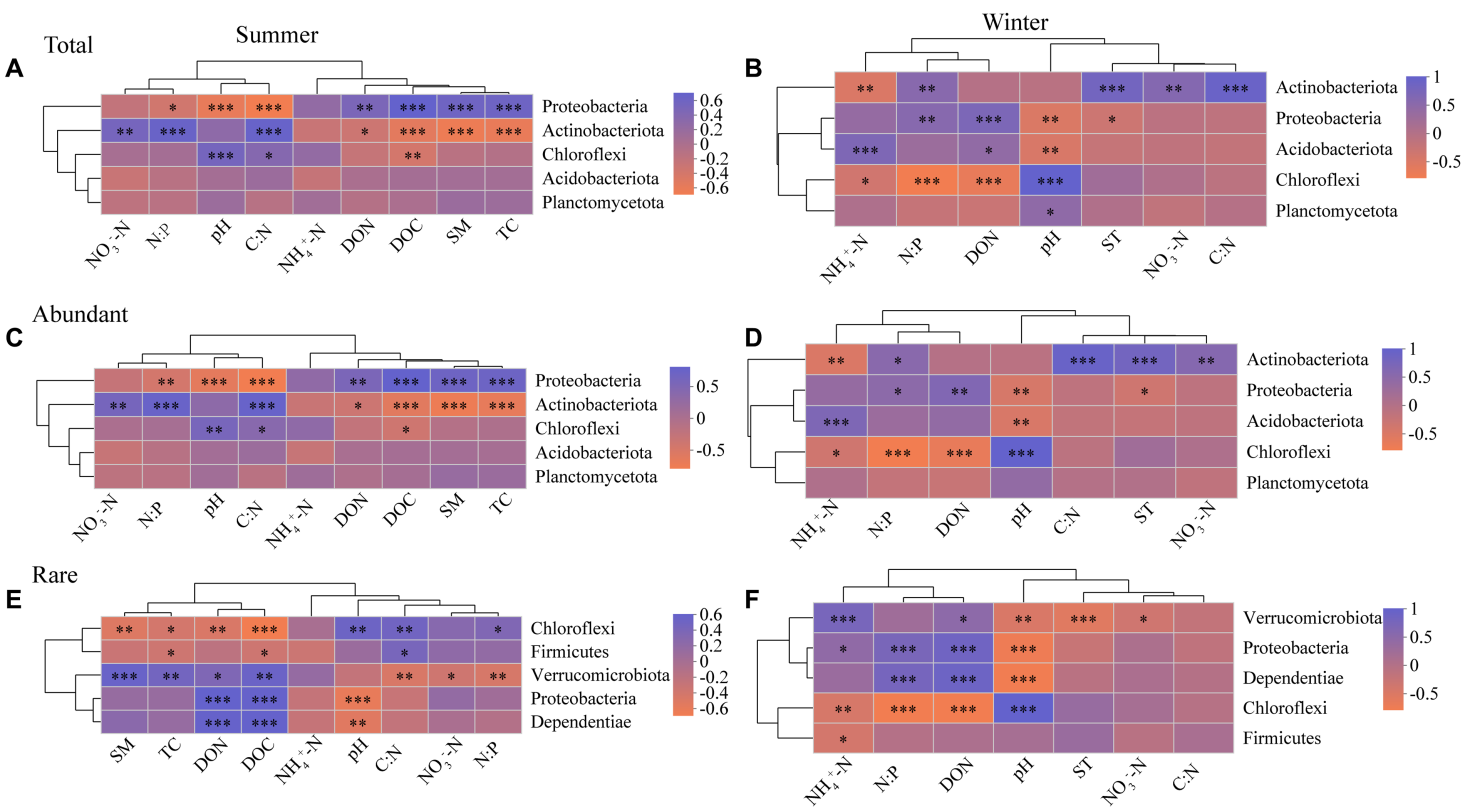
Spearman correlation heatmap showing the relationship between soil physico-chemical properties and the top 5 most abundant taxa in the total **(A)**, abundant **(C)**, and rare **(E)** bacterial communities in summer, and the total **(B)**, abundant **(D)**, and rare **(F)** bacterial communities in winter, respectively (*n* = 80). *p ≤ 0.05, **p ≤ 0.01, ***p ≤ 0.001. Red indicates a negative correlation, blue indicates a positive correlation, and the color interval of different *R* values is displayed on the scale on the right side. Abbreviations for soil physico-chemical properties are shown in Table 1.

For in summer, Proteobacteria of abundant bacterial community has more environmental associations than rare bacteria, whereas Chloroflexi of rare bacterial community has more environmental associations than abundant bacteria (Figures 4C,E). For in winter, however, the impact of soil physico-chemical properties on Proteobacteria and Chloroflexi of abundant bacteria are similar with rare bacteria (Figures 4D,F). Proteobacteria and Chloroflexi showed significant negative and positive correlation with soil pH, respectively (Figure 4). Soil NH_4_^+^-N had no significant effect on the top 5 most abundant taxa in the abundant and rare bacterial communities in summer (Figures 4C,E), while significantly affecting them in winter (Figures 4D,F). NO_3_^−^-N was positively correlated with Actinobacteriota of abundant bacteria, and negatively correlated with Verrucomicrobiota of rare bacteria (Figure 4).

### 3.4 Interaction network of abundant and rare bacterial community across seasons

**Figure 5:**
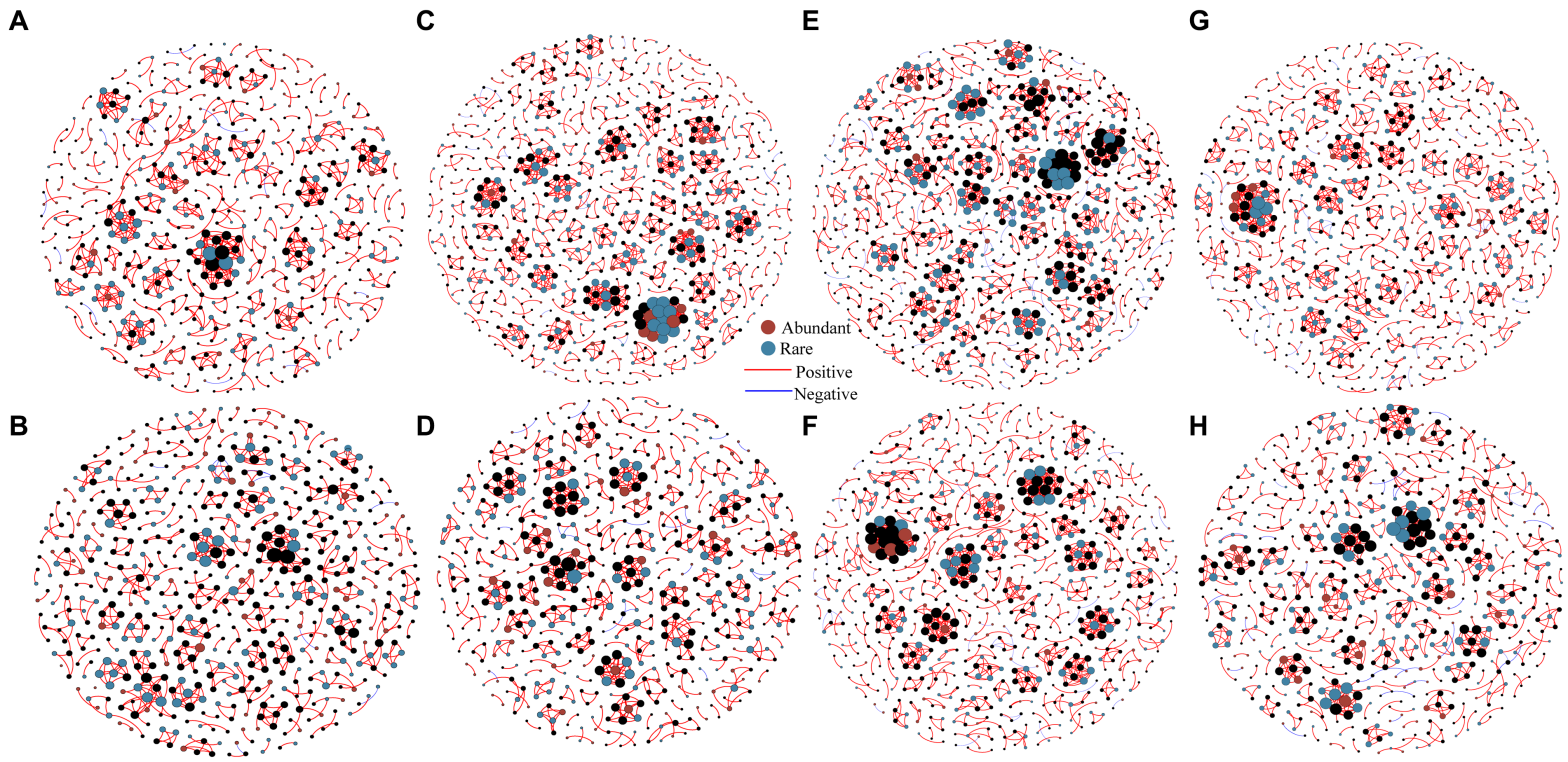
Co-occurrence networks of soil bacteria at 430 m **(A)**, 1,200 m **(C)**, 1700 m **(E)**, 2,100 m **(G)** in summer, and at 430 m **(B)**, 1,200 m **(D)**, 1700 m **(F)**, 2,100 m **(H)** in winter were investigated by network analysis (*n* = 80). The connection demonstrates a robust correlation (Spearman’s *r* > 0.8) that is statistically significant (*p* < 0.01). Topological properties of networks were shown in Supplementary Table S11.

Spearman correlation coefficients were employed to generate interaction network between abundant and rare bacterial communities at each elevational gradient during summer and winter to investigate co-occurrence patterns (Figure 5). Here, the majority of nodes were classified as rare bacteria, while only few nodes were categorized as abundant bacteria in both the summer and winter at each elevational gradient (Supplementary Table S10). Notably, most connections (abundant-rare, abundant-abundant and rare-rare taxa) between OTUs had positive correlations (Supplementary Table S11). Comparing with the topological properties of the network level, it can be found that the nodes, links, average degree and clustering coefficient of summer network at each elevational gradient were greater than in the winter network, indicating that the connectivity in summer was higher, compared with winter, and the relationship was closer (Supplementary Table S10). In general, the nodes and links of rare bacteria are higher than abundant bacteria at each elevational gradient in summer and winter (Supplementary Tables S10, S11), indicating that rare bacterial composition was more complex than abundant bacteria. Microbial networks at each elevational gradient were distinctly different, the connections at 1700 m were higher than other elevational gradients (Supplementary Table S10), which suggested a complex and stable bacterial community at 1700 m (Figure 5 and Supplementary Table S10).

The topological roles of nodes are discriminated using Zi-Pi plots (Supplementary Figure S4). Nodes assigned to module hubs, network hubs and connectors are generalists, which were considered as the key organisms in microbial communities (Montoya et al., 2006). According to the topological roles of the eight networks, a total of 751 connectors were identified in summer networks, including 335 abundant species and 416 rare species, and 641 connectors were identified in winter networks, including 310 abundant species and 331 rare species (Figure 5). In general, connectors were observed in both summer and winter bacterial networks, only one rare bacterial node (OTU5859) classified as a modular hub at 1700 m in summer, and no network hubs were observed.

## 4 Discussion

### 4.1 Distribution patterns of abundant and rare bacterial diversity, community and co-occurrence along elevational gradients and seasons

The study revealed distinct patterns in abundant and rare bacterial diversity along elevational gradient in subtropical mountain ecosystems, which is harmony with the outcome of Li et al. (2017). Interestingly, despite the differences in diversity patterns, both these two communities displayed similar responses to the elevational gradient in summer and winter. Generally, total and abundant bacterial diversity first decreased and then increased with elevation, and the lowest α-diversity was observed at 1700 m. In contrast, rare bacterial α-diversity were highest at 1700 m (Figure 1 and Supplementary Table S3). Together, the diversity patterns of both these two bacterial communities varies response to environmental changes along the elevational gradient, thereby supporting our initial hypothesis. Environment has an essential function in shaping the abundance and distribution patterns. Persistent environmental changes have distinct effects on these two groups, leading to different response (Pedrós-Alió, 2012; Jiao et al., 2019). Temperature, moisture and nutrient availability in temperate desert systems are associated with higher richness of abundant bacteria, while resulting in a decline of rare bacteria (Wang et al., 2021a). In present study, DOC and DON showed the highest values at 1700 m elevation (Supplementary Table S2). Interestingly, we found contrasting relationship between these soil factors and abundant and rare bacterial diversity (Supplementary Table S5). Specifically, we discovered that abundant bacterial diversity was negatively associated with the levels of DOC and DON. In contrast, we observed a positive correlation between the rare bacterial diversity and the levels of DOC and DON.

Elevation plays a pivotal role in practically shaping soil microbial bacterial communities (Singh D. et al., 2014; Tian et al., 2023). Elevational gradients exerted a substantial impact on abundant and rare bacterial communities, thereby demonstrating a consistent response of these communities to the elevational gradient (Figure 2 and Supplementary Table S6). This may be explained by similarity in soil physico-chemical properties, which exerted comparable effects on these communities (Figure 3 and Supplementary Tables S8, S9). However, elevational gradients and seasons explained 54.88% variation of abundant bacterial community; while only 8.57% variation of rare bacterial community were explained (*p* < 0.01, Figure 2). Hence, our findings suggest that these two communities exhibit similar responses to changes induced by elevations and seasons, but have divergent intensive responses. Specifically, the abundant bacterial community appears to be more strongly influenced by environmental variables along elevational gradients and across seasons. The result of MST ratio also indicated abundant bacterial communities was more strongly driven by deterministic assembly processes in summer and winter (MST < 50%), while stochastic processes had a greater impact on rare bacterial community (MST > 50%) (Supplementary Figure S3). This observation aligns with previous studies in soil ecosystems, which have shown that deterministic processes related to environmental factors governed abundant bacterial communities. In contrast, the influences on rare bacterial communities are predominantly governed by stochastic processes, such as geographical distance (Hou et al., 2020).

All OTUs in abundant bacterial communities could be classified, while 5.18% of OTUs in rare bacterial communities could not be classified (Supplementary Figure S1). Indeed, our current understanding and expertise in microbial ecology are predominantly concentrates on abundant taxa, primarily owing to their higher relative abundance and easier detection in environmental samples. However, it is important to acknowledge that low-abundance rare microorganisms, despite their lower population sizes, play critical ecological roles and may have significant impacts on ecosystem functioning (Lynch and Neufeld, 2015). Rare species have more connections and nodes in ecological networks compared to abundant taxa (Figure 5 and Supplementary Table S11), indicating their potential importance in maintaining network stability and functioning. This suggests that rare taxa may serve as key species, contributing significantly to the overall network structure and functioning (Jiao et al., 2017). As shown by earlier research, nitrifying archaea with low abundance played a decisive function in the nitrogen biogeochemical cycle (Bahram et al., 2022). Indeed, rare bacterial communities, with their complex phyla composition, perform a paramount role in microbial diversity and contribute to ecosystem stabilization (Supplementary Figure S1). Their broad taxonomic diversity enhances functional redundancy and resilience to environmental disturbances, ensuring the continuity of important ecological processes (Xu et al., 2022).

Microorganisms engage in diverse ecological relationships, such as mutualism and competition, which contribute to the formation of complex interaction networks (Faust and Raes, 2012). In our study, the relationship between abundant and rare bacteria was predominantly positive (Supplementary Table S10), indicating a cooperative interaction between these groups (Ju et al., 2014). The frequent cooperation observed within both abundant and rare bacterial communities can strengthen their adaptability in the face of a changing environment. The presence of interaction networks among microbes can provide a buffer, allowing them to better cope with extreme environmental events. These networks facilitate the overall stability and adaptability of microbial communities (Konopka et al., 2015). Moreover, the quantity of positive connections among bacteria was greater in summer compared to winter (Supplementary Tables S10, S11). This suggests that bacteria exhibit stronger resistance and resilience to environmental disturbances during the summer season. Additionally, the number of co-occurring network nodes and links was found to be higher at high elevations compared to low elevations (Supplementary Table S10). This discovery aligns with earlier researches that have stated a greater abundance of network links and nodes at higher elevations (Ma et al., 2016).

### 4.2 Impact of soil physico-chemical properties on the abundant and rare bacterial community and diversity

Abundant and rare bacterial diversity were markedly associated with soil pH, indicating that pH had some effect on microbial diversity (Supplementary Table S5). This finding aligns with earlier research indicating the considerable contribution of pH on the overall soil bacterial community (Wang Y. et al., 2017; Wang et al., 2021b). Additionally, soil bacterial diversity can be well predicted by changes in temperature (Zhou et al., 2016). However, soil temperature was only a significant factor of abundant bacterial diversity rather than rare bacterial diversity (Supplementary Table S5). This observation suggests that rare bacteria may possess adaptive mechanisms enabling them to tolerate and adapt to environment temperature variations (Wilson and Walker, 2010). Rare bacterial diversity was dramatically associated with NH_4_^+^-N compared to soil abundant bacterial diversity (Supplementary Table S5). A possible explanation for the observed patterns is that species in ecological systems may occupy specific ecological niches that are associated with the availability of nitrogen (Harpole and Tilman, 2007). It is likely that abundant and rare species coexist within the ecosystem by occupying distinct ecological niches that provide them with unique resource requirements and environmental conditions. This niche differentiation could contribute to the maintenance of abundant and rare species in the ecosystem. Rare taxa demonstrate a significant function in maintaining microbial diversity (Jiao et al., 2019). Rare bacterial diversity is highly responsive to even minor alterations in the soil environment (He J. et al., 2023). Observed the relative abundance and diversity of rare taxa were more susceptible to varying soil physicochemical properties along the elevational gradient than abundant taxa (Supplementary Table S5 and Figure 4). Our results support the notion that abundant taxa exhibit greater environmental resistance, while rare bacterial diversity is more sensitive to variations in the soil environment. Even a small negative change in the abundance of rare bacteria can have a disproportionate impact and potentially lead to their extinction (Pedrós-Alió, 2006).

Soil properties were the foremost determinants influencing regional and global soil microbial community patterns (Ma et al., 2015; He L. et al., 2023). Thus, quantifying the contribution of soil physico-chemical properties to shaping abundant and rare microbial communities is required. The distance decay theory, which asserts that the similarity of communities decreases as geographic distance increases, is a fundamental model in ecology (Soininen et al., 2007). Elevation-distance decay relationships are observed in both abundant and rare bacterial communities (Zhou et al., 2022). However, the abundant bacterial communities exhibit higher elevation-distance decay rates compared to rare bacteria (Li et al., 2017). This observation suggests that abundant bacterial communities may be more sensitive to elevation-related changes in soil properties than rare bacterial communities. Our results support this finding, as soil physico-chemical properties explained approximately 59.11 and 56.02% variation of the abundant bacterial community at summer and winter, respectively. The sensitivity of abundant bacterial communities to changes in soil factors induced by elevation gradients is believed to be higher. By contrast, only 6.95 and 7.41% of rare bacterial community variations could be explained (Figure 3). The composition and dynamics of the rare bacterial community may be influenced to a greater extent by other factors (e.g., stochastic processes), rather than by environmental factors such as soil physico-chemical properties (Li et al., 2017). Seasonal changes directly impact soil properties, which in turn influence the microbial community (Buscardo et al., 2018). Rare bacterial community are more connected with soil N:P, whereas soil moisture was the principal variable governing the difference of abundant bacterial communities in summer (Supplementary Table S8). However, soil temperature and pH play the predominant determinants in determining the elevational difference in winter, respectively (Supplementary Table S9). The results imply that soil physico-chemical properties made different contributions to the abundant and rare bacterial communities in the different season. This concurs with earlier study conducted by Su et al. (2022), which also observed distinct responses of these two communities to soil properties. First, soil physical properties, especially soil water content and temperature may be predictors of the seasonal changes of bacterial communities (Berg et al., 1998). Similarly, previous reports have emphasized the explicit contribution of soil pH in driving seasonal variations in bacterial community composition (Buscardo et al., 2018). This study further contributes to this understanding by highlighting the differential factors influencing the seasonal changes of abundant and rare bacterial communities in subtropical mountain ecosystem.

Soil physico-chemical properties are major factor in shaping top 5 most abundant taxa in these two communities (Figure 4). Some Proteobacteria is an autotrophic organism, which can utilize inorganic carbon sources for growth (Fierer et al., 2007). This explains why the Proteobacteria is positively correlated with the dissolved organic nitrogen content of the soil. Acidobacteriota, on the other hand, is considered to be a group of oligotrophic bacteria that are adapted to low-nutrient environments. They are often found to be more abundant in soils with low pH, suggesting a preference for acidic conditions (Fierer et al., 2007). The distinct ecological characteristics of Proteobacteria and Acidobacteriota contribute to their different responses to soil nutrient and pH conditions. The increase of pH and the decrease of C and N content are main reasons for the increase of Chloroflexi (Liu et al., 2020), which indicates a positive correlation between abundance of Chloroflexi and soil pH, as well as a negative correlation with soil nutrient levels (Figure 4). These observations highlight the response of Chloroflexi to variations in soil properties, particularly pH and nutrient availability.

## 5 Conclusion

In this study, we conducted an investigation into the elevational patterns and drivers of the both abundant and rare bacterial communities in different seasons. Our findings revealed abundant and rare bacterial communities respond similarly to elevational gradients; however, the elevational patterns of diversity were contrasting. Abundant bacterial diversity showed a U-shaped elevational pattern, while rare bacterial diversity increased with elevation. Soil properties played a prominent role in shaping the elevational patterns of abundant bacterial communities. Additionally, rare taxa, characterized by a larger number of nodes and connections in network, appeared to be of particular ecological importance. Overall, these findings expand upon existing knowledge regarding the elevational patterns of rare and abundant bacterial diversity and offer valuable insights into the ecological significance of these taxa.

## Data availability statement

The datasets presented in this study can be found in online repositories. The names of the repository/repositories and accession number(s) can be found in the article/Supplementary material.

## Author contributions

PW: Investigation, Visualization, Writing – original draft, Writing – review & editing, Conceptualization. DH: Conceptualization, Funding acquisition, Writing – review & editing. JG: Investigation, Writing – review & editing. JL: Investigation, Visualization, Writing – review & editing. QZ: Conceptualization, Writing – review & editing. DC: Conceptualization, Funding acquisition, Writing – review & editing.

## Footnotes

^1^www.majorbio.com
