# Supplementary material for: Unraveling the spatial–temporal distribution patterns of soil abundant and rare bacterial communities in China’s subtropical mountain forest
Source: Front Microbiol. 2024 Feb 12;15:1323887. doi: 10.3389/fmicb.2024.1323887 (PMC10895375; doi:10.3389/fmicb.2024.1323887)
Supplement: Supplementary file 1 [file Data_Sheet_1.docx]

# Supplementary and methods

## DNA extraction and sequencing

Soil genomic DNA were extracted using the DNeasy^®^ PowerSoil® Pro Kit (QIAGEN, USA) following the manufacturer’s instructions. The quality assessment of DNA extracts was examined using 1% agarose gel electrophoresis. The concentration and purity of the DNA extracts were determined using a NanoDrop 2000 UV-Vis spectrophotometer (Thermo Scientific, Wilmington, USA). For amplifying the hypervariable region V3-V4 of the 16S rRNA gene, the forward primer 338F (5'-ACTCCTACGGGAGGCAGCAG-3') and reverse primer 806R(5'-GGACTACHVGGGTWTCTAAT-3') were used. The PCR mixtures contained 5 × *TransStart* FastPfu buffer 4 μL, 2.5 mM dNTPs 2 μL, forward primer (5 μM) 0.8 μL, reverse primer (5 μM) 0.8 μL, *TransStart* FastPfu DNA Polymerase 0.4 μL, template DNA 10 ng, and ddH_2_O up to 20 μL. The amplification conditions were as follows: 95℃ initial denaturation for 3min, 27 cycles (95℃ denaturation for 30s, 55℃ annealing for 30s, 72℃ extension for 45s), then 72℃ stable extension for 10min, finally stored at 10℃. The purified PCR products were quantified using a Quantus™ Fluorometer (Promega, USA). Purified amplicons were employed for library construction using NEXTFLEX
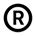
 Rapid DNA-Seq Kit and paired-end sequenced was performed using Illumina MiSeq PE300 platform (Illumina, San Diego, USA) at Majorbio Bio-Pharm Technology Co. Ltd. (Shanghai, China). The raw sequencing data were upload to the NCBI Sequence Read Archive (SRA) database (Accession Number: SRP437273).

## Processing of sequencing data

The raw 16S rRNA gene sequencing reads were demultiplexed, quality-filtered by fastp version 0.20.0 (Chen et al., 2018) and merged by FLASH version 1.2.7 (Magoč and Salzberg, 2011) with the following criteria: (i) the 300 bp reads were truncated at any site receiving an average quality score of <20 over a 50 bp sliding window, and the truncated reads shorter than 50 bp were discarded, reads containing ambiguous characters were also discarded; (ii) only overlapping sequences longer than 10 bp were assembled according to their overlapped sequence. The maximum mismatch ratio of overlap region is 0.2. Reads that could not be assembled were discarded; (iii) Samples were distinguished according to the barcode and primers, and the sequence direction was adjusted, exact barcode matching, 2 nucleotide mismatches in primer matching. UPARSE 7.1 was performed to cluster into operational taxonomic units (OTUs) with a 97% similarity (Stackebrandt and Goebel, 1994; Edgar, 2013) and chimeric sequences were identified and removed. The RDP Classifier 2.2 (Wang et al., 2007) was used for OTU representative sequence classification analysis against 16S rRNA database (Silva v138) setting confidence threshold of 0.7. A total of 4,965,869 high-quality sequences representing bacterial communities were identified across all samples. These sequences were further clustered into 8,935 OTUs based on a 97% sequence similarity threshold.

# Supplementary tables and figures

Table S1 Results for the effects of elevation, season and their interactions on soil physico-chemical properties using linear mixed effects model (n=80).

| Soil physicochemical characteristics | Elevation | | Seasons | | Elevation×Season | |
| --- | --- | --- | --- | --- | --- | --- |
|  | F | P | F | P | F | P |
| SM（%） | 223.6829 | **<0.001** | 14.5784 | **<0.001** | 5.1448 | **<0.05** |
| ST (°C) | 437.01 | **<0.001** | 14506.43 | **<0.001** | 266.89 | **<0.001** |
| pH | 3.827 | 0.0601 | 3.639 | 0.0640 | 1.721 | 0.1974 |
| NH_4_^+^-N（mg·kg^-1^） | 49.9958 | **<0.001** | 13.3906 | **<0.001** | 0.7166 | 0.4026 |
| NO_3_^-^-N（mg·kg^-1^） | 33.31690 | **<0.001** | 13.79760 | **<0.001** | 19.57032 | **<0.001** |
| TC（g·kg^-1^） | 42.7127 | **<0.001** | 1.5785 | 0.2166 | 4.2670 | **<0.05** |
| TN（g·kg^-1^） | 113.3671 | **<0.001** | 3.4703 | 0.0702 | 7.0012 | **<0.05** |
| TP（g·kg^-1^） | 204.0140 | **<0.001** | 2.3475 | 0.1338 | 27.6292 | **<0.001** |
| C:N | 53.074 | **<0.001** | 0.548 | 0.4638 | 1.825 | 0.1847 |
| C:P | 87.7294 | **<0.001** | 11.1505 | **<0.01** | 18.7054 | **<0.001** |
| N:P | 33.1003 | **<0.001** | 16.4824 | **<0.001** | 18.9051 | **<0.001** |
| DON（mg·kg^-1^） | 3.56530 | 0.0690 | 0.45994 | 0.5018 | 0.36436 | 0.5497 |
| DOC（mg·kg^-1^） | 5.26755 | **<0.05** | 2.53976 | 0.1193 | 0.57546 | 0.4528 |

Note: Abbreviations of soil physico-chemical properties are defined in Table 1.

Table S2 The one-way ANOVA and t-tests showing elevational and seasonal differences in soil physico-chemical properties (Data were shown as mean ± SE, n=10).

| Soil physico-chemical properties | Summer | | | | Winter | | | |
| --- | --- | --- | --- | --- | --- | --- | --- | --- |
|  | 430 m | 1200 m | 1700 m | 2100 m | 430 m | 1200 m | 1700 m | 2100 m |
| SM (%) | **14.80±1.30cB** | **24.81±0.88bB** | **42.14±3.03a** | **45.23±1.44a** | **19.49±0.91cA** | **41.13±1.34bA** | **45.94±0.77a** | **42.02±1.27b** |
| pH | **4.69±0.07a** | **4.48±0.05bA** | **3.96±0.06cB** | **4.60±0.06ab** | **4.82±0.03a** | **4.18±0.04bB** | **4.44±0.04cA** | **4.73±0.04a** |
| ST (°C) | **24.35±0.01aA** | **20.46±0.02bA** | **19.05±0.16cA** | **18.86±0.07cA** | **7.29±0.02aB** | **7.13±0.02bB** | **7.01±0.04cB** | **6.52±0.06dB** |
| NH_4_^+^-N (mg·kg^-1^) | **31.28±4.47bcA** | **21.20±1.81cB** | **38.68±2.43bA** | **54.20±4.96aA** | **7.07±1.44cB** | **30.89±3.68abA** | **29.54±1.88bB** | **37.97±3.48aB** |
| NO_3_^-^-N (mg·kg^-1^) | **10.42±2.32aA** | **0.77±0.20b** | **2.56±0.71b** | **0.07±0.02b** | **1.56±0.35aB** | **0.86±0.40ab** | **0.99±0.19a** | **0.08±0.02b** |
| TC (g·kg^-1^) | **31.79±1.48cA** | **41.68±2.66bB** | **57.04±1.52aA** | **64.32±5.18aA** | **24.50±1.89cB** | **64.14±5.13aA** | **47.24±0.87bB** | **44.29±1.74bB** |
| TN (g·kg^-1^) | **1.76±0.07cA** | **2.42±0.15bB** | **4.39±0.11aA** | **4.42±0.32aA** | **1.44±0.09bB** | **3.58±0.28aA** | **3.57±0.09aB** | **3.16±0.14aB** |
| TP (g·kg^-1^) | **0.13±0.005dB** | **0.23±0.01cB** | **0.40±0.01bA** | **0.70±0.11aA** | **0.17±0.006dA** | **0.31±0.02cA** | **0.36±0.01bB** | **0.55±0.02aB** |
| C:N | **18.07±0.32aA** | **17.21±0.26b** | **12.99±0.21d** | **14.50±0.19c** | **16.96±0.40bB** | **17.90±0.31a** | **13.28±0.26c** | **14.04±0.18c** |
| C:P | **238.86±7.62aA** | **179.64±7.83bB** | **143.90±5.54c** | **91.98±4.67dA** | **143.21±7.08bB** | **206.07±7.79aA** | **133.69±4.75b** | **81.05±2.21cB** |
| N:P | **13.20±0.28aA** | **10.41±0.34bB** | **11.05±0.30bA** | **6.34±0.29c** | **8.41±0.28cB** | **11.49±0.33aA** | **10.05±0.24bB** | **5.78±0.16d** |
| DON (mg·kg^-1^) | **6.25±0.80bA** | **8.79±0.86bB** | **19.88±2.26a** | **8.13±1.24bA** | **4.20±0.43bB** | **17.21±1.60aA** | **19.71±2.11a** | **5.08±0.53bB** |
| DOC (mg·kg^-1^) | **16.91±0.73cA** | **25.12±2.71bB** | **51.75±2.65a** | **27.71±3.13bA** | **9.04±1.10dB** | **70.31±5.03aA** | **49.12±1.86b** | **18.50±1.77cB** |

Note: Uppercase letters indicate significant differences between seasons for the same elevation and lowercase letters indicate significant differences among elevational gradinent for the same season. *, significant at *P* < 0.05. Abbreviations of soil physico-chemical properties are defined in Table 1.

Table S3 The One-way ANOVA and t-tests showing elevational and seasonal differences in Shannon and Simpson index of soil bacterial community (Data were shown as mean ± SE, n=10).

|  | | Summer | | | | Winter | | | |
| --- | --- | --- | --- | --- | --- | --- | --- | --- | --- |
|  |  | 430 m | 1200 m | 1700 m | 2100 m | 430 m | 1200 m | 1700 m | 2100 m |
| Total bacteria | Shannon | 5.76±0.07a | 5.78±0.05aA | 5.52±0.04b | 5.78±0.05a | 5.78±0.05a | 5.55±0.07bB | 5.51±0.09b | 5.81±0.06a |
|  | Simpson | 0.010±0.0009b | 0.012±0.0007b | 0.015±0.0009a | 0.011±0.0009b | 0.011±0.0009a | 0.013±0.0012a | 0.015±0.0014a | 0.011±0.0007a |
| Abundant bacteria | Shannon | 5.44±0.05a | 5.40±0.04aA | 5.17±0.04b | 5.40±0.04a | 5.39±0.04a | 5.18±0.06bB | 5.16±0.07b | 5.40±0.04a |
|  | Simpson | 0.012±0.0009b | 0.014±0.0008b | 0.017±0.001a | 0.013±0.0009b | 0.013±0.001a | 0.016±0.001a | 0.017±0.002a | 0.013±0.0008a |
| Rare bacteria | Shannon | 5.10±0.02b | 5.20±0.01a | 5.23±0.007aA | 5.21±0.008a | 5.11±0.01b | 5.17±0.02a | 5.19±0.02aB | 5.18±0.01a |
|  | Simpson | 0.002±0.0001a | 0.001±0.00007b | 0.0008±0.00005bB | 0.0009±0.00006b | 0.0017±0.0001a | 0.0012±0.0001b | 0.0011±0.0001bA | 0.0012±0.0001b |

Note: Uppercase letters indicate significant differences between seasons for the same elevation and lowercase letters indicate significant differences among elevational gradient for the same season. *, significant at *P* < 0.05.

Table S4 Results for the effects of elevation, season and their interactions on total, abundant and rare bacterial α-diversities using linear mixed effects model (n=80).

|  | | Elevation effect | | Season effect | | Elevation × Season | |
| --- | --- | --- | --- | --- | --- | --- | --- |
|  |  | F | P | F | P | F | P |
| Total bacteria | Shannon | 0.68 | 0.4175 | 1.02 | 0.3189 | 0.11 | 0.7382 |
|  | Simpson | 2.1902 | 0.1497 | 0.2297 | 0.6345 | 0.6131 | 0.4385 |
| Abundant bacteria | Shannon | 1.95 | 0.1732 | 3.15 | 0.0840 | 0.68 | 0.4163 |
|  | Simpson | 2.4471 | 0.1286 | 0.4819 | 0.4918 | 1.0062 | 0.3222 |
| Rare bacteria | Shannon | 38.8 | **<0.001** | 6.1 | **<0.05** | 3.0 | 0.0930 |
|  | Simpson | 38.1899 | **<0.001** | 6.6125 | **<0.05** | 3.4191 | 0.0722 |

Table S5 The pearson correlation analysis showing the relationship between total, abundant and rare bacterial diversity and soil physico-chemical properties (n=80).

| Factors | Total bacterial diversity | | Abundant bacterial diversity | | Rare bacterial diversity | |
| --- | --- | --- | --- | --- | --- | --- |
|  | Shannon | Simpson | Shannon | Simpson | Shannon | Simpson |
| ST (°C) | 0.133 | -0.101 | **0.223*** | -0.128 | 0.060 | -0.066 |
| SM (%) | **-0.283*** | **0.275*** | **-0.357**** | **0.281*** | **0.518**** | **-0.520**** |
| pH | **0.509**** | **-0.400**** | **0.527**** | **-0.384**** | **-0.302**** | **0.306**** |
| NH_4_^+^-N (mg·kg^-1^) | -0.046 | 0.035 | -0.039 | 0.024 | **0.407**** | **-0.417**** |
| NO_3_^-^-N (mg·kg^-1^) | 0.084 | -0.218 | 0.172 | **-0.255*** | **-0.366**** | **0.362**** |
| TC (g·kg^-1^) | **-0.296**** | **0.239*** | **-0.337**** | **0.238*** | **0.419**** | **-0.427**** |
| TN (g·kg^-1^) | **-0.310**** | **0.299**** | **-0.358**** | **0.299**** | **0.541**** | **-0.546**** |
| TP (g·kg^-1^) | 0.019 | 0.047 | -0.030 | 0.054 | **0.473**** | **-0.476**** |
| C:N | 0.154 | **-0.250*** | 0.201 | **-0.256*** | **-0.498**** | **0.492**** |
| C:P | -0.128 | -0.020 | -0.059 | -0.044 | **-0.360**** | **0.354**** |
| N:P | **-0.284*** | 0.146 | **-0.221*** | 0.119 | -0.194 | 0.191 |
| DON (mg·kg^-1^) | **-0.501**** | **0.462**** | **-0.524**** | **0.447**** | **0.344**** | **-0.339**** |
| DOC (mg·kg^-1^) | **-0.551**** | **0.435**** | **-0.588**** | **0.427**** | **0.265*** | **-0.269*** |

Note: Abbreviations of soil physico-chemical properties are defined in Table 1.

Table S6 Analysis of similarities (ANOSIM) showing the elevational differences in the total, abundant and rare bacterial communities (n=80).

| Elevation | Total bacterial community | | Abundant bacterial community | | Rare bacterial community | |
| --- | --- | --- | --- | --- | --- | --- |
|  | summer | winter | summer | winter | summer | winter |
| 430 m vs 1200 m | **0.001***** | **0.001***** | **0.001***** | **0.001***** | **0.001***** | **0.001***** |
| 430 m vs 1700 m | **0.001***** | **0.001***** | **0.001***** | **0.001***** | **0.001***** | **0.001***** |
| 430 m vs 2100 m | **0.001***** | **0.001***** | **0.001***** | **0.001***** | **0.001***** | **0.001***** |
| 1200 m vs 1700 m | **0.001***** | **0.001***** | **0.001***** | **0.001***** | **0.001***** | **0.001***** |
| 1200 m vs 2100 m | **0.001***** | **0.001***** | **0.001***** | **0.001***** | **0.001***** | **0.001***** |
| 1700 m vs 2100 m | **0.001***** | **0.001***** | **0.001***** | **0.001***** | **0.001***** | **0.001***** |

Table S7 Analysis of similarities (ANOSIM) showing the seasonal differences in the total, abundant and rare bacterial communities (n=80).

| Elevation | Total bacterial community | Abundant bacterial community | Rare bacterial community |
| --- | --- | --- | --- |
|  | Summer vs winter | Summer vs winter | Summer vs winter |
| 430 m | **0.001***** | **0.001***** | **0.001***** |
| 1200 m | **0.001***** | **0.001***** | **0.001***** |
| 1700 m | **0.001***** | **0.001***** | **0.019*** |
| 2100 m | **0.001***** | **0.001***** | 0.241 |

Table S8 The permutest analysis showing relationship between soil physico-chemical properties and total, abundant and rare bacterial communities in soil in summer (n=80).

| Factors | Total bacterial community in summer | | Abundant bacterial community in summer | | Rare bacterial community in summer | |
| --- | --- | --- | --- | --- | --- | --- |
|  | R^2^ | P | R^2^ | P | R^2^ | P |
| SM (%) | 0.8389 | **0.001** | 0.8368 | **0.001** | 0.7688 | **0.001** |
| pH | 0.6732 | **0.001** | 0.6748 | **0.001** | 0.5371 | **0.001** |
| NH_4_^+^-N (mg·kg^-1^) | 0.4576 | **0.001** | 0.4579 | **0.001** | 0.4634 | **0.001** |
| NO_3_^-^-N (mg·kg^-1^) | 0.3119 | **0.003** | 0.314 | **0.003** | 0.5071 | **0.001** |
| TC (g·kg^-1^) | 0.6932 | **0.001** | 0.6894 | **0.001** | 0.561 | **0.001** |
| C:N | 0.7881 | **0.001** | 0.7904 | **0.001** | 0.6811 | **0.001** |
| N:P | 0.7153 | **0.001** | 0.7133 | **0.001** | 0.8094 | **0.001** |
| DON (mg·kg^-1^) | 0.5946 | **0.001** | 0.5956 | **0.001** | 0.404 | **0.001** |
| DOC (mg·kg^-1^) | 0.7574 | **0.001** | 0.7617 | **0.001** | 0.5339 | **0.001** |

Note: Abbreviations of soil physico-chemical properties are defined in Table 1.

Table S9 The permutest analysis showing relationship between soil physico-chemical properties and total, abundant and rare bacterial communities in soil in winter (n=80).

| Factors | Total bacterial community in winter | | Abundant bacterial community in winter | | Rare bacterial community in winter | |
| --- | --- | --- | --- | --- | --- | --- |
|  | R^2^ | P | R^2^ | P | R^2^ | P |
| ST (°C) | 0.8005 | **0.001** | 0.8034 | **0.001** | 0.8598 | **0.001** |
| pH | 0.4516 | **0.001** | 0.4644 | **0.001** | 0.884 | **0.001** |
| NH_4_^+^-N (mg·kg^-1^) | 0.6656 | **0.001** | 0.6642 | **0.001** | 0.7477 | **0.001** |
| NO_3_^-^-N (mg·kg^-1^) | 0.1785 | **0.024** | 0.1816 | **0.018** | 0.3249 | **0.002** |
| C:N | 0.4604 | **0.001** | 0.4555 | **0.001** | 0.4829 | **0.001** |
| N:P | 0.6752 | **0.001** | 0.6835 | **0.001** | 0.9154 | **0.001** |
| DON (mg·kg^-1^) | 0.6756 | **0.001** | 0.6829 | **0.001** | 0.5931 | **0.001** |

Note: Abbreviations of soil physico-chemical properties are defined in Table 1.

Table S10 Topological properties of co-occurring abundant-rare taxa networks obtained in summer and winter was identified by network analysis (n=80).

| Network metrics | 430 m | | 1200 m | | 1700 m | | 2100 m | |
| --- | --- | --- | --- | --- | --- | --- | --- | --- |
|  | Summer | Winter | Summer | Winter | Summer | Winter | Summer | Winter |
| Number of nodes | 599  (112, 159) | 579  (80, 151) | 810  (95, 259) | 596  (92, 171) | 839  (99,284) | 758 (126,184) | 759  (106, 268) | 634  (89, 181) |
| Number of edges | 843 | 704 | 1393 | 816 | 1399 | 1224 | 1134 | 869 |
| Number of positive correlations | 831 | 692 | 1376 | 797 | 1349 | 1192 | 1101 | 840 |
| Number of negative correlations | 12 | 12 | 17 | 19 | 50 | 32 | 33 | 29 |
| Average clustering coefficient | 0.842 | 0.844 | 0.87 | 0.856 | 0.866 | 0.84 | 0.853 | 0.872 |
| Average path length | 1.299 | 1.239 | 1.213 | 1.224 | 1.176 | 1.197 | 1.217 | 1.155 |
| Network diameter | 4 | 3 | 3 | 4 | 3 | 4 | 4 | 3 |
| Average degree | 2.815 | 2.432 | 3.44 | 2.738 | 3.335 | 3.23 | 2.988 | 2.741 |
| Modularity | 0.987 | 1.001 | 0.985 | 1.006 | 1.019 | 0.998 | 1.005 | 1.015 |

Note: Number of abundant and rare taxa is in parentheses, respectively.

Table S11 A summary of associations between each subcommunity under different seasons was identified by network analysis (n=80).

|  | | Total connections | | Positive connections | | Negative connections | |
| --- | --- | --- | --- | --- | --- | --- | --- |
|  |  | Summer | Winter | Summer | Winter | Summer | Winter |
| 430 m | Abundant-Abundant | 24 | 18 | 20 | 17 | 4 | 1 |
|  | Abundant-Rare | 25 | 11 | 25 | 11 | 0 | 0 |
|  | Rare-Rare | 111 | 76 | 110 | 76 | 1 | 0 |
| 1200 m | Abundant-Abundant | 29 | 26 | 26 | 23 | 3 | 3 |
|  | Abundant-Rare | 51 | 19 | 51 | 19 | 0 | 0 |
|  | Rare-Rare | 199 | 71 | 198 | 66 | 1 | 5 |
| 1700 m | Abundant-Abundant | 26 | 41 | 16 | 30 | 10 | 11 |
|  | Abundant-Rare | 34 | 18 | 31 | 18 | 3 | 0 |
|  | Rare-Rare | 231 | 117 | 225 | 117 | 6 | 0 |
| 2100 m | Abundant-Abundant | 44 | 31 | 29 | 20 | 15 | 11 |
|  | Abundant-Rare | 11 | 8 | 10 | 8 | 1 | 0 |
|  | Rare-Rare | 195 | 102 | 192 | 99 | 3 | 3 |


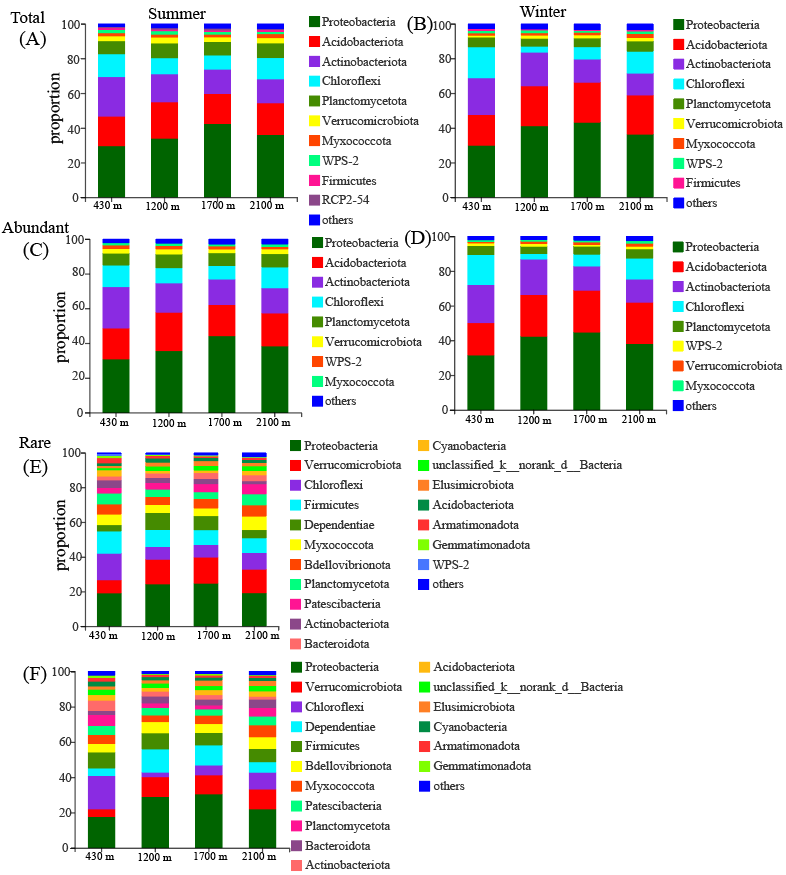


**FIGURE S1** Community barplot analysis showing Phyla compositions of abundant and rare bacterial communities between different elevations and seasons (n=80). Compositions of total (A, B), abundant (C, D) and rare (E, F) bacterial communities between different elevations and seasons.


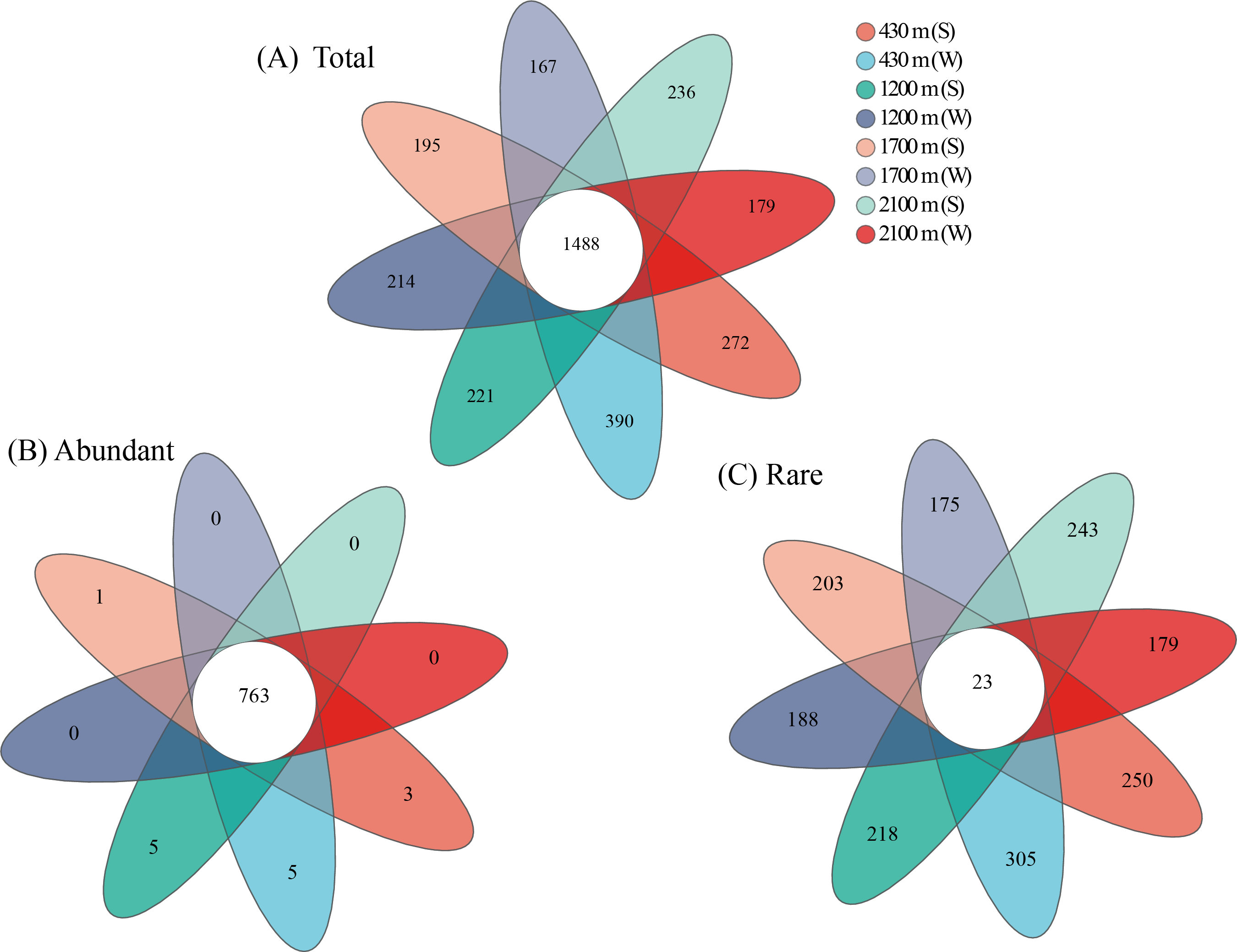


**FIGURE S2.** Venn plot showing the shared and unique OTUs of total (A), abundant (B) and rare (C) bacterial taxa in soils at 430 m, 1200 m, 1700 m and 2100 m elevation in summer and winter, respectively (n=80). The number of unique species to each group is shown in the petals, while the number of shared species to all groups is shown in the center.


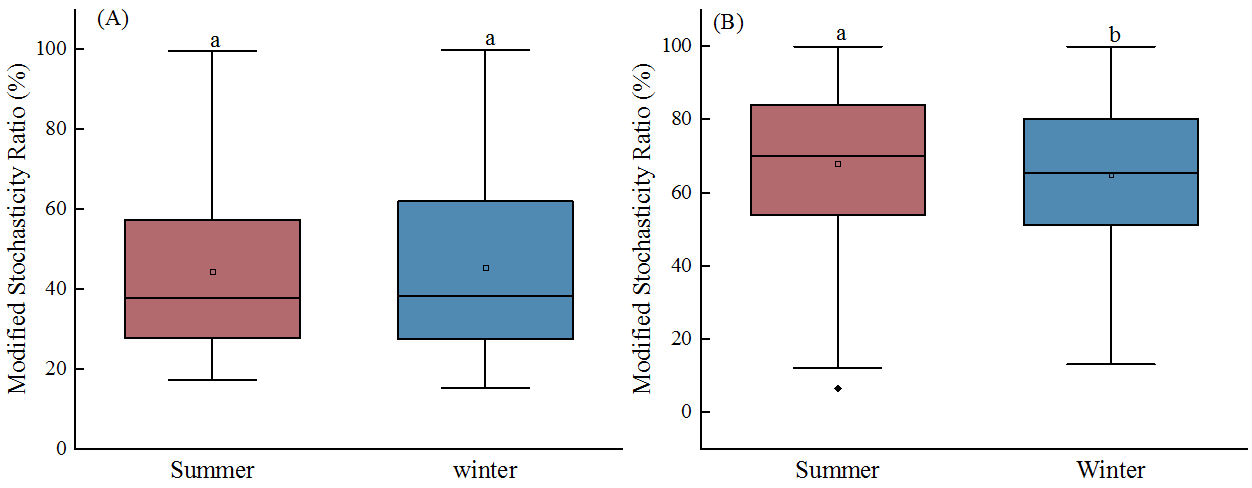


**FIGURE S3** Modified normalized stochasticity (MST) ratio of (a) abundant bacterial community and (b) rare bacterial community (n=80). Different letters indicate significant differences between in summer and winter (*P* < 0.05).


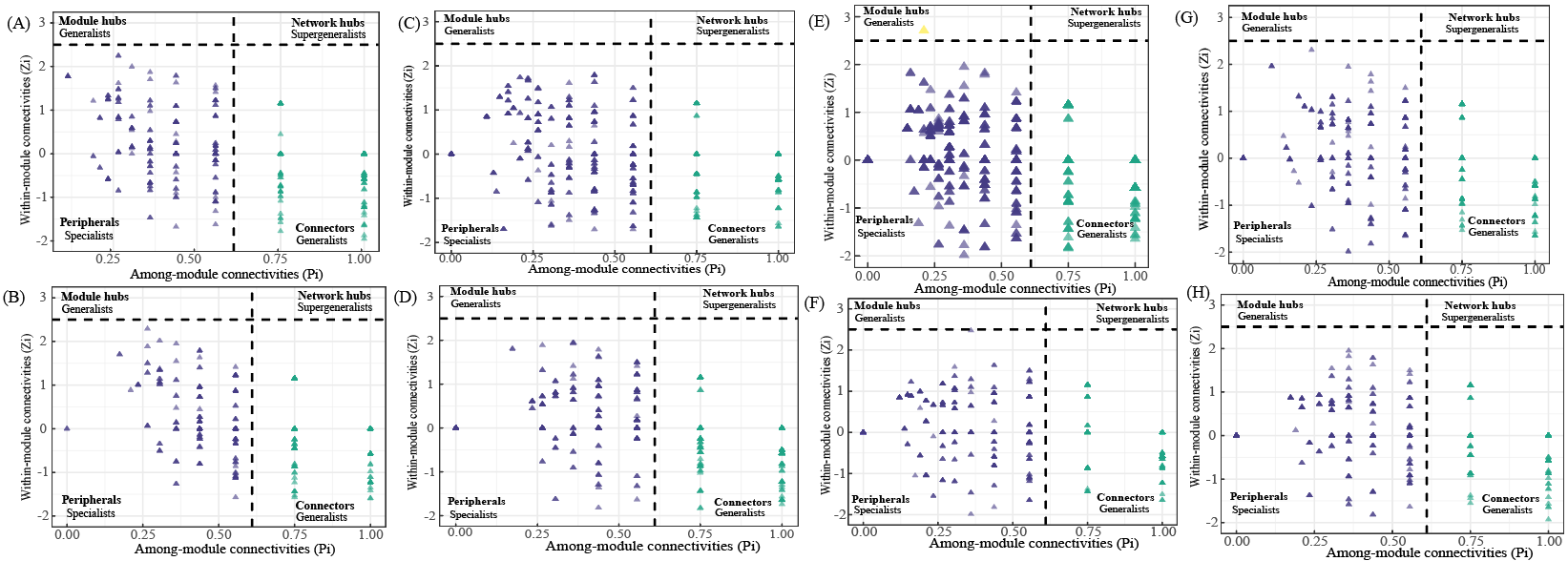


**FIGURE S4** Zi- Pi plots of soil bacterial at 430 m (A), 1200 m (C), 1700 m (E), 2100 m (G) in summer, and at 430 m (B), 1200 m (D), 1700 m (F), 2100 m (H) in winter based on OTUs topological roles in bacterial networks (n=80).

# References

Chen, S., Zhou, Y., Chen, Y., Gu, J., 2018. fastp: an ultra-fast all-in-one FASTQ preprocessor. Bioinformatics. 34, i884-i890. https://doi.org/10.1093/bioinformatics/bty560.

Edgar, R.C., 2013. UPARSE: highly accurate OTU sequences from microbial amplicon reads. Nat. Methods. 10, 996-998. https://doi.org/10.1038/nmeth.2604.

Magoč, T., Salzberg, S.L., 2011. FLASH: fast length adjustment of short reads to improve genome assemblies. Bioinformatics. 27, 2957-2963. https://doi.org/10.1093/bioinformatics/btr507.

Stackebrandt, E., Goebel, B.M., 1994. Taxonomic note: a place for DNA-DNA reassociation and 16S rRNA sequence analysis in the present species definition in bacteriology. Int. J. Syst. Evol. Microbiol. 44, 846-849. https://doi.org/10.1099/00207713-44-4-846.

Wang, Q., Garrity, G.M., Tiedje, J.M., Cole, J.R., 2007. Naive Bayesian classifier for rapid assignment of rRNA sequences into the new bacterial taxonomy. Appl. Environ. Microbiol. 73, 5261-5267. https://doi.org/10.1128/AEM.00062-07.
